# The Association Between the Flesh Colour and Carotenoid Profile of 25 Cultivars of Mangoes

**DOI:** 10.3390/molecules30081661

**Published:** 2025-04-08

**Authors:** Tatsuyoshi Takagi, Hung Hong, Natalie Dillon, Peter Crisp, Daniel Cozzolino, Tim O’Hare

**Affiliations:** 1 Centre of Nutrition and Food Science, Queensland Alliance of Agriculture and Food Innovation (QAAFI), The University of Queensland, Brisbane, QLD 4072, Australia; d.cozzolino@uq.edu.au (D.C.); t.ohare@uq.edu.au (T.O.); 2 School of Agriculture and Food Sustainability, The University of Queensland, Brisbane, QLD 4072, Australia; h.trieu@uq.edu.au (H.H.); p.crisp@uq.edu.au (P.C.); 3 Department of Primary Industries, Mareeba, QLD 4880, Australia; natalie.dillon@daf.qld.gov.au

**Keywords:** outer and inner pigmentation, pulp colour, β-carotene, mesocarp

## Abstract

Mango (*Mangifera indica* L.) cultivars display a wide range of ripe flesh colours, from deep-orange to pale-yellow, largely linked to differences in carotenoid profiles. This study examined the relationship between carotenoid profile and flesh colour across 25 mango cultivars. Flesh colour was measured using chroma (intensity) and hue angle (colour) and correlated with carotenoid content quantified using ultra-high performance liquid chromatography with diode-array detection (UHPLC-DAD). Chroma and hue angle displayed a second-order inverse polynomial relationship with chroma increasing as hue angle decreased. At lower hue angles, a wider range of chroma values was observed. Regression analysis showed that chroma correlated most strongly with total carotenoid concentration (TCC) (R^2^ = 0.54), followed by total orange carotenoid concentration (R^2^ = 0.47) and all-*trans* β-carotene concentration (R^2^ = 0.45). Hue angle correlated most strongly with the total orange carotenoid concentration (R^2^ = 0.76), then all-*trans* β-carotene concentration (R^2^ = 0.73) and TCC (R^2^ = 0.64). Interestingly, pale-yellow cultivars often exhibited low carotenoid concentrations, high titratable acidity and low soluble solids, suggesting incomplete ripening may also contribute to their pale colouration. These results provide insight into carotenoid-colour relationships and offer direction for phytochemical-based breeding programmes targeting mango flesh colour traits.

## 1. Introduction

Mango (*Mangifera indica* L.) cultivars vary in flesh colour when ripe, ranging from deep-dark-orange to pale-yellow. This variability in flesh colour has been reported in various mango cultivars to be largely linked to a difference in carotenoid profile [1,2]. In scientific literature, flesh colour is often objectively measured using the Commission Internationale de l’Eclairage (CIE) L*C*h colour system, splitting colour attributes into lightness (L), chroma (C) and hue angle (h) [3]. Useful metrics in measuring mango flesh colour are chroma and hue angle, with chroma being a measure of colour intensity (i.e., pale versus dark) and hue angle a measure of colour (i.e., yellow versus orange). In mangoes, the relevant range of hue angle for ripe flesh is approximately 70° to 95°, with orange colouration represented by lower angles to a more yellow colouration at higher hue angles [3,4,5]. In fully ripened mangoes, chroma and hue angle have been reported to range from 48.9 to 67.4, and 74.4° to 93.4°, respectively; although, these values can vary depending on the ripening conditions and method of colour measurement [5,6].

The carotenoid profile is known to have a significant impact on the flesh colour, and in all mango cultivars to date, the principal carotenoid has been reported as all-*trans* β-carotene [7,8]. All-*trans* β-carotene is an orange-pigmented carotenoid [9], well known for its role in being a major dietary precursor for Vitamin A [10]. In addition, other carotenoids have been identified, including the yellow carotenoids, α-carotene, lutein and luteoxanthin, as well as other orange carotenoids such as zeaxanthin, β-cryptoxanthin and violaxanthin [7,11,12,13,14,15]. The differences in colour among carotenoids are primarily due to variations in their chromophores, specifically, the length of their conjugated double-bond system. A longer chain of conjugated double-bonds shift light absorption toward longer wavelengths, resulting in deeper-orange to red hues, while shorter chains absorb at shorter wavelengths, producing more yellow colouration [16,17].

In addition to the various all-*trans* carotenoids mentioned above, *cis*-isomers of β-carotene have also been reported in significant amounts. The predominant *cis*-isomers include 9-*cis* and 13-*cis* β-carotene, both of which are associated with yellow colouration and have been identified in multiple cultivars of mangoes [18,19]. Vásquez-Caicedo et al. (2005) reported that the *cis*-to-*trans* isomer ratio can range from 14% to 40% [19]. This isomerisation may contribute to mango flesh colour variation, as *cis*-isomers absorb light differently from their trans counterparts due to shortening of the chromophore [16,20].

Moreover, higher total carotenoid concentrations (TCC) have been associated with more intensely coloured yellow-orange cultivars compared to those with paler or less vibrant pigmentation [19]. Factors influencing TCC can include the efficiency of key enzymes in the carotenoid biosynthesis pathway, such as phytoene synthase [21]. In addition, the size and development of chromoplasts, which are the organelles where carotenoids are synthesised and stored, can significantly impact final carotenoid accumulation and ultimately affect flesh colour [22,23].

The relationship between the carotenoid profile and both flesh colour and intensity has yet to be fully established in mangoes and, in this respect, this study was conducted to quantify the relationship between of the mentioned features (as determined by objective measurement of hue angle and chroma) with the cultivar carotenoid profile. It is expected that flesh colour is associated with the total concentration of orange carotenoids and the colour intensity more closely correlated with the total carotenoid concentration.

## 2. Materials and Methods

### 2.1. Fruit Collection and Ripening

Fruit from 24 mango cultivars were harvested at a hard-mature green stage from the Queensland Department of Agriculture and Fisheries (DAF) mango germplasm collection, located at the Walkamin Research Facility (Cairns, Queensland, Australia). Cultivar selection was spread across pale-yellow to dark-orange flesh colour, based on previously recorded subjective measurements of each cultivar’s flesh colour when fully ripe (Department of Agriculture and Fisheries, 2024). The colour descriptions utilised five flesh colour codes/cards with the following descriptions for each code: dark-orange (1), orange (2), orange-yellow (3), yellow (4) and green/pale-yellow (5). The colour codes were based on colour cards selected from the Royal Horticultural Society colour charts [24], to ensure consistency in subjective assessment across seasons and between assessors. One additional cultivar (cv. Calypso^®^) which is popular in Australia was also obtained from a Brisbane supermarket, as it was not available at the time of harvest.

The collected fruits were ripened at 23 °C after initially being submerged in a 1000 ppm Ethrel^®^ solution for 3 min to synchronise the initiation of the ripening process. Full ripeness of the mango fruit, which occurred between 7 and 14 days after treatment with Ethrel^®^ was primarily gauged on the firmness of the pulp, as measured by hand. Additional indicators, such as the production of a strong aroma and changes in skin colour, were also used as secondary indicators of full ripeness. The six most closely synchronised fruits from each cultivar were selected as replicates from an initial 10 fruit ripened for ongoing analysis.

### 2.2. Sample Preparation and Colour Assessment

Two mango cheeks from either side of the central large seed of each fully ripened replicate were severed, followed by the separation of the flesh from the skin using a spoon. The flesh was roughly diced and one half of the flesh blended (Westinghouse Model WHSM04SS, Australia) to create a homogenised sample for objective colour measurement (hue and chroma). A CR-400 chromameter (Konica Minolta, Osaka, Japan) was utilised to obtain the readings of chroma and hue angle, with each value being an average of three readings. The remaining half of the flesh sample was stored in a freezer (−35 °C) prior to freeze-drying for subsequent carotenoid extraction. Once dry, the freeze-dried samples were blended using a Commercial Laboratory Blender (Waring, Stamford, CT, USA) until finely powdered.

### 2.3. Titratable Acidity (TA) and Total Solubel Solids (TSS)

Titratable acidity (TA) and total soluble solids (TSS) were conducted on the homogenised samples used for colour measurement. TA was measured using a 765 Dosimat (Metrohm, Gladesville, Australia) connected to a 632 pH-Meter (Metrohm, Australia) and 614 Impulsomat (Metrohm, Australia). TSS was measured using a pocket refractometer (65th anniversary edition) (ATAGO, Tokyo, Japan). Results were expressed as % citric acid equivalents and °Brix for TA and TSS, respectively.

### 2.4. Carotenoid Extraction and Analysis

Carotenoid extraction was based on the method by Hong et al. [25], with minor modifications. All extraction steps were performed in a dimly lit environment to prevent light-induced carotenoid degradation. Six biological replicates from each cultivar were analysed in triplicate (three technical replicates). Approximately 1 g of freeze-dried flesh powder was placed into a 50 mL Falcon^®^ tube, to which a 2 mL mixture of methyl tert-butyl (MTBE): methanol (MeOH) (1:1) with a 0.1% butylated hydroxytoluene (BHT) (*w*/*v*) and 1 mL of methanolic potassium hydroxide (15%) (KOH) was added for saponification.

The tubes were wrapped in aluminium foil before being placed on an orbital shaker at 2 OPM × 100 (reciprocating platform model RP1812, (Paton Scientific, Adelaide, Australia) for one hour. Sodium phosphate buffer (pH 3) and extraction solution consisting of hexane:dichloromethane (DCM) (7:3) was subsequently added, homogenised using an Ultra Turrax T 25 Basic (Ika WERKE, Guangzhou, China), and placed on the orbital shaker for 10 min. The samples were then centrifuged (Eppendorf centrifuge 5810, Hamburg, Germany) for 5 min at 4000 rpm and the supernatants transferred into a new Falcon^®^ tube and the process repeated until the bottom pellet was devoid of colour.

The combined supernatants were dried under nitrogen gas. The MTBE:MeOH:BHT mixture (0.95 mL) was added to rehydrate the evaporated product and an internal standard of *trans-*β-apo-8’-carotenal (0.05 mL, 100 ppm) was added before filtering the solution with a 0.22 µm syringe filter into a 2 mL vial for immediate carotenoid analysis. Carotenoid analysis was conducted using ultra-high performance liquid chromatography coupled with diode-array detection (UHPLC-DAD) (Shimadzu, Kyoto, Japan), as described by Hong et al. [25].

Carotenoids were quantified as β-carotene equivalents, using a β-carotene reference standard for all carotenoids, except lutein, which was quantified using a reference lutein standard. All standards utilised were purchased from Sapphire Bioscience Pty Ltd. (Waterloo, Australia). β2 and β3 detected in this study are predicted to be the minor isomers of β-carotene such as 7-*cis* and 11-*cis*.

### 2.5. Ultra-High Performance Liquid Chromatography Coupled with Diode-Array Detection (UHPLC-DAD)

Carotenoid quantification was conducted using a UHPLC-DAD (Shimadzu, Kyoto, Japan), consisting of a system controller (SCL-40), diode-array detector (SPD-M30A), autosampler (SIL-40CX3), three pumps (LC-40D X3), two degassers (DGU-405) and a column heater (CTO-40C). LabSolutions software (Version 5.85 Shimadzu) was utilised to record the UV/Vis spectra in the 170–700 nm range, and the carotenoids were viewed at 450 nm wavelength.

A C30 YMC carotenoid column (5 µm, 250 × 4.6 mm, YMC, Kinesis, Redland Bay, Australia) was utilised for chromatographic separation. Flow rate was set to 0.6 mL/min and the gradient was set to 95% of mobile phase A (90% MeOH, 8% MTBE, 2% RO and 0.1% formic acid) and 5% mobile phase B (90% MTBE, 8% MeOH, 2% RO and 0.1% formic acid). Mobile phase A was decreased to 75% at 20 min, followed by a further decrease to 30% at 30 min before being increased to 95% at 35 min until completion. Column oven temperature was maintained at 25 °C, and a minimum and maximum pressure of 200 psi and 3800 psi was set. The solvents and chemicals utilised in this study were purchased either from Sigma-Aldrich or Merek (Darmstadt, Germany).

### 2.6. Statistical Analysis

All statistical analyses were performed using R (version 4.3.2). One-way analysis of variance (ANOVA) followed by least significant difference (LSD) tests (*p* < 0.05) were conducted to assess differences between mango cultivars. Variables were standardised prior to analysis by scaling to unit variance (1/SD).

Principal Component Analysis (PCA) was conducted on standardised data to assess variation in mango colour and other measured data. The Partial Least Squares (PLS) regression was performed also on the same standardised data to examine the relationships between carotenoid concentrations and mango colour attributes (hue and chroma). The leave-one-out cross-validation (LOO-CV) was utilised to determine optimal number of components. All data visualisations, including PCA and PLS plots, were generated in R. Regression curves were created using Microsoft Excel.

## 3. Results

### 3.1. Colour and Intensity

**Figure 1:**
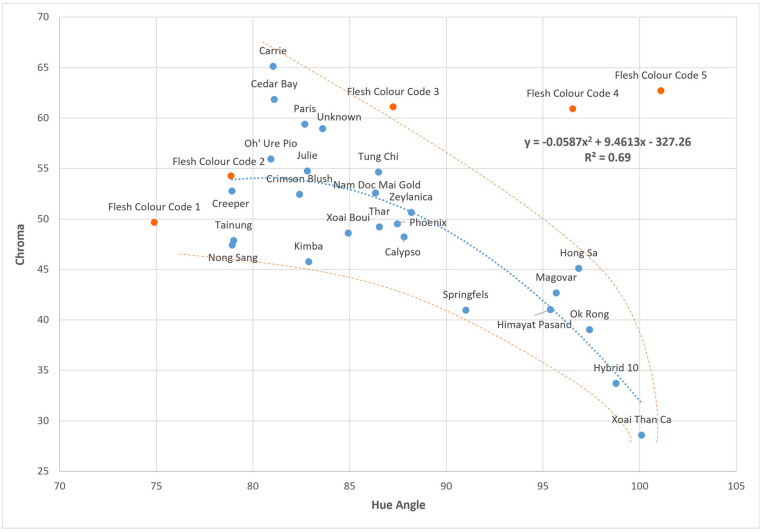
Average flesh hue angle and chroma values of 25 cultivars of fully ripe mangoes (blue circles, n = 6). The comparative values of the five flesh colour cards utilised to categorise the mango colours are shown as orange circles. The above and below orange dotted lines indicate the general spread of the cultivars. A second-order inverse polynomial trend line for all cultivars was further fitted in blue with an equation of y = −0.0587x^2^ + 9.4613x − 327.26.

The 25 cultivars of mangoes investigated in this study were plotted using their hue angle and chroma values (Figure 1). Additionally, the hue angle and chroma values of the five flesh colour code cards were plotted and demonstrated to have an even spread along the hue angle axis, but not along the chroma axis.

The highest hue angle (most yellow) was observed in cv. Xoai Than Ca with a hue angle of 100.1°. This was also paired with the lowest chroma of 28.6, as observed in the bottom right of Figure 1. At the other end of the range, the lowest hue angle (most orange) was observed in cv. Creeper, with a hue angle of 78.9° and a chroma of 52.8. The highest chroma was observed with cv. Carrie, consisting of a chroma of 65.1, but with a higher hue angle of 81.0°.

The chroma and hue angle were observed to exhibit a second-order inverse polynomial correlation, with chroma generally increasing as hue angle decreased. As such, orange-fleshed cultivars (lower hue angle) tended to have a higher intensity of colour (higher chroma). This relationship was observed to have a moderate R^2^ (coefficient of determination) of 0.69. From Figure 1, however, it appeared that there was a spread in the range of chroma as hue angle decreased, forming a horn-like shape, as seen by the above and below orange-dotted lines (Figure 1).

### 3.2. Carotenoid Profiling

**Figure 2:**
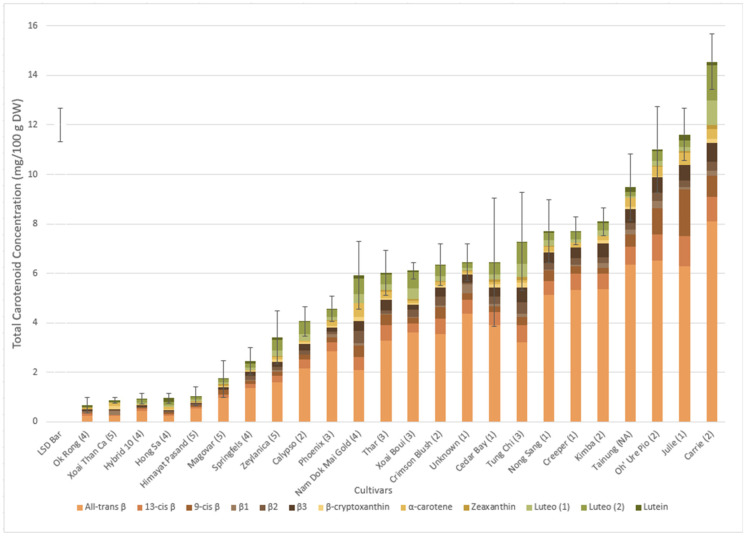
Total carotenoid concentration (TCC) (mg/100 g DW) of each cultivar using six biological replicates and three technical replicates. The subjective colour code for each cultivar is also included in the brackets (1–5). The average proportions in DW are also visualised for each cultivar with each carotenoid having their respective colour as per the legend. The error bars represent one standard deviation above and below the mean for each cultivar of mango. A vertical LSD bar (1.37) is displayed in the leftmost column to indicate the least significant difference.

The total carotenoid concentration (TCC) and carotenoid profiles of the 25 cultivars measured in their dry weight (DW) form are shown in Figure 2. TCC ranged from 0.66 to 14.55 mg/100 g DW, with the highest TCC observed with cv. Carrie, which also showed the highest chroma value (Figure 1). By contrast, the lowest TCC was observed in cv. Ok Rong. Interestingly, the lowest TCC was not recorded in cv. Xoai Than Ca, despite displaying the highest hue angle and lowest chroma (Figure 1), however the five cultivars with the lowest TCC were not statistically different according to the Fishers’ protected LSD test. Generally, the TCC of the individual cultivars increased according to the 1–5 flesh colour codes of green/pale-yellow (5) through to dark-orange (1), with increasing concentration of carotenoids observed in the more intense orange cultivars, or as hue angle decreased (Figure 2).

Figure 2 also depicts the individual carotenoid profiles of the 25 cultivars. The major carotenoids included all-*trans* β-carotene, α-carotene, lutein, zeaxanthin, β-cryptoxanthin, isomers of luteoxanthin and *cis*-isomers of β-carotene (including predicted isomers β2 and β3). Violaxanthin, which was detected in other studies, was not observed.

All-*trans* β-carotene was identified as being the principal carotenoid in all cultivars, having the highest concentration in cv. Carrie (8.1 mg/100 g DW), and the lowest concentration in cv. Xoai Than Ca (0.23 mg/100 g DW) (Figure 2). In fact, cv. Xoai Than Ca showed the lowest concentration in multiple carotenoids, such as 13-*cis* β-carotene, 9-*cis* β-carotene and both luteoxanthin isomers.

The yellow cv. Nam Dok Mai Gold had the highest concentrations in α-carotene and in one of the predicted β-carotene isomers (labelled as β2). Generally, cultivars belonging to the dark-orange category displayed the highest concentrations of all individual carotenoids; therefore cv. Nam Dok Mai Gold (a yellow cultivar) was an exception to this observation. Interestingly, other yellow and green/pale-yellow cultivars (flesh colour code 4 and 5) also possessed high concentrations of these two yellow carotenoids. In general, cultivars which had been previously subjectively labelled as ‘5’ (green/pale-yellow) were found to have the lowest concentrations of each carotenoid.

### 3.3. Proportions of Carotenoids

The 25 mango cultivars varied in their proportions of individual carotenoids (Table 1). For all cultivars, all-*trans* β-carotene was the highest proportional carotenoid, ranging from 68% in orange-fleshed cv. Creeper to 25% in the yellow-fleshed cv. Hong Sa.

The most interesting profile of the proportions was observed in cvs. Xoai Than Ca and Hong Sa. Due to the low proportions of the principal carotenoid, all-*trans* β-carotene, the minor carotenoids were in much higher proportions than in the other cultivars.

Regarding α-carotene, cv. Xoai Than Ca, Hong Sa and Nam Dok Mai Gold were significantly higher than the rest of the cultivars with 13.07%, 9.39% and 9.25%, respectively. The other cultivars only had a proportion of up to 5% at most. This trend of pale-yellow and yellow cultivars having high proportions of other yellow carotenoids such as β-carotene isomer 2 and lutein were consistent.

### 3.4. Correlation Between Colour Intensity (Chroma) and Carotenoid Profile

**Figure 3:**
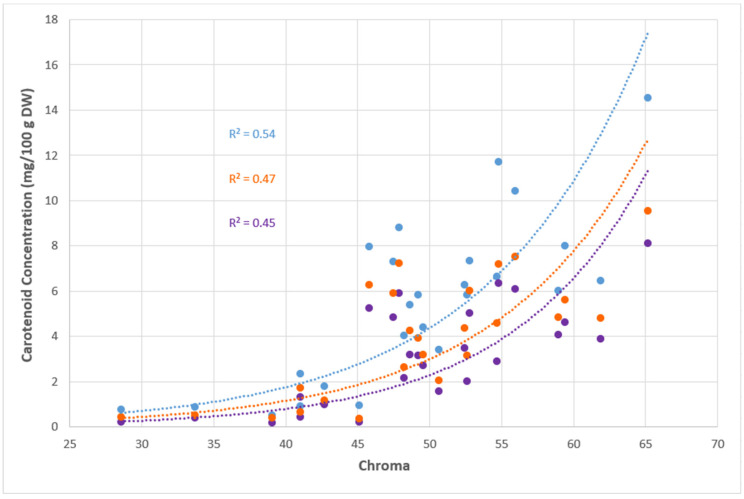
Total carotenoid concentration (TCC) (mg/100 g DW) (blue circles), all-*trans* β-carotene concentration (mg/100 g DW) (purple circles) and sum of orange carotenoid concentrations (orange circles) of each cultivar against their respective chroma values are indicated. The TCC, orange carotenoids and all-*trans* β-carotene concentration are the average of six biological replicates.

A positive exponential relationship between TCC and chroma (R^2^ = 0.54) was observed (Figure 3). The coefficient of determination (R^2^) decreased to 0.47 when chroma was correlated with the sum of all orange carotenoids and further declined to 0.45 when chroma was only correlated with the principal orange carotenoid, all-*trans* β-carotene. The orange carotenoids consisted of β-cryptoxanthin, β-carotene isomer 2, β-carotene isomer 3, all-*trans* β-carotene and zeaxanthin. Figure 3 demonstrates that the quantity of carotenoids holds a large impact on the chroma, which measures the intensity of colour, rather than the individual carotenoid profiles.

### 3.5. Correlation Between Colour (Hue Angle) and Carotenoid Profile

**Figure 4:**
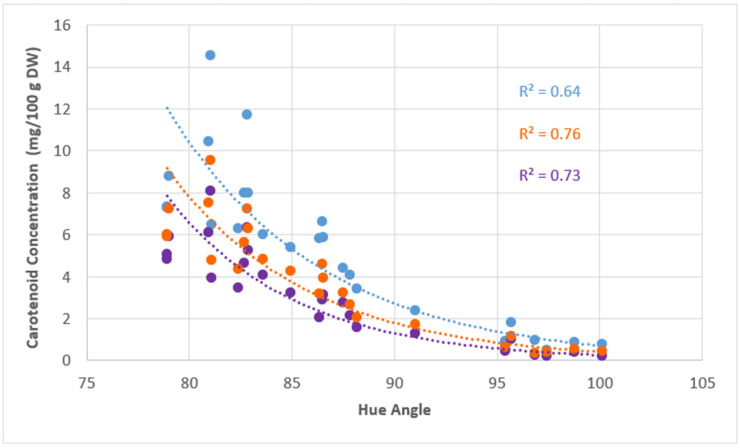
The total carotenoid concentration (TCC) (mg/100 g DW) (blue circles), sum of orange carotenoid concentrations (orange circles) and all-*trans* β-carotene concentration (purple circles), against their respective hue angles are indicated. The sum of orange carotenoids, all-*trans* β-carotene concentration and TCC are the average of six biological replicates.

A strong inverse exponential relationship (R^2^ = 0.76) between the sum of the concentration of orange carotenoids and hue angle was observed (Figure 4), with hue angle decreasing as the sum of the concentration of orange carotenoids increased. The strength of the correlation (R^2^) declined slightly to 0.73 when only the principal orange carotenoid, all-*trans* β-carotene, was correlated with hue angle, and further declined to 0.64 when TCC (which includes all orange and yellow carotenoids) was correlated. Figure 4 would, therefore, indicate that orange carotenoid concentration, rather than TCC has greater influence over hue angle.

### 3.6. Titratable Acidity (TA) and Total Soluble Solids (TSS)

TA and TSS were assessed using the homogenised sample after objectively measuring the flesh colour. The TA ranged from 0.11 g/100 g in cv. Xoai Boui to as high as 0.69 g/100 g in cv. Xoai Than Ca, but the average was 0.23 g/100 g. The lowest TSS was identified in cv. Xoai Than Ca (9.82 g/100 g) whilst the highest occurred in cv. Paris (20.63 g/100 g), resulting in an average of 15.62 g/100 g.

Differences among cultivars were evident across all three measurements, as detailed in Table 2. The LSD values for TA of 0.06 and 2.1 for TSS indicate significant variability among cultivars. The TSS did not exhibit any consistent trends across cultivars, with most cultivars falling within the LSD range, indicating no significant differences in sweetness. In contrast, the TA values showed that cultivars with green/pale-yellow flesh generally measured the highest acidity level.

### 3.7. Principal Component Analysis (PCA) and Partial Least Square (PLS) Regression

A principal component analysis (PCA) was conducted using carotenoid concentration, TSS, TA and colour measurements of each cultivar, based on the averages of six biological replicates (Figure 1). The first principal component (PC1) accounted for 37% of the variance in the dataset, while the second principal component (PC2) accounted for 14%.

PC1 was largely represented by the concentrations of carotenoids and colour measurements. In the negative region of PC1, variables such as TCC and chroma were strongly represented, while hue was predominant in the positive region. PC2, on the other hand, was largely influenced by the proportions of carotenoids. The proportion of yellow carotenoids was strongly positive, whilst the orange carotenoid proportion was strongly negative with the TCC located centrally.

All individual carotenoid concentrations were situated in the negative region of PC1, indicating their positive association with TCC. In contrast, the proportions of individual carotenoids, labelled as ‘percent ‘carotenoid’’ differed between the yellow and orange carotenoids. Proportions of orange carotenoids, such as all-*trans* β-carotene, β-cryptoxanthin, zeaxanthin, β-carotene isomers 2 and 3 were associated with orange-coloured cultivars in the negative region of PC1. Conversely, proportions of yellow carotenoids, such as lutein, α-carotene, β-carotene isomer 1, 13-*cis* β-carotene, 9-*cis* β-carotene and luteoxanthin isomers 1 and 2 were linked to yellow-coloured cultivars in the positive region of PC1.

The PCA revealed that some cultivars exhibited unique positioning amongst the other cultivars. The cv. Nam Dok Mai Gold was centrally located on PC2 but had a strongly positive loading on PC1, with no other cultivar sharing a similar placement. On the other hand, cvs. Unknown and Creeper were positioned opposite to cv. Nam Dok Mai Gold, indicating differentiation along PC1 but not along PC2. On the PC2 axis, cv. Carrie was positioned strongly negative, whereas cvs. Hong Sa and Xoai Than Ca were strongly positive, highlighting the clear separation.

TA was associated with higher hue angles and proportions of yellow carotenoids, aligning with the positive region of PC1. Meanwhile, TSS was positioned in the upper left quadrant of the PCA plot, demonstrating an association with higher chroma and higher carotenoid concentrations.

Partial least squares (PLS) regression was performed to examine the relationship between hue angle and chroma and their respective associations with TCC, carotenoid compositions, TSS, and TA (Table 3). For the PLS model predicting hue angle, the first component explained 52.3% of the variance with an additional 11.6% captured by the second component. TCC (−0.49), total orange carotenoid concentration (TOCC) (−0.51) and total orange carotenoid proportion (TOCP) (−0.39) had the strongest negative loadings on the first PLS component, while total yellow carotenoid proportion (TYCP) (0.39) displayed a positive association with hue angle.

In the PLS model predicting chroma, the first component explained 48.9% of the predictor variance, with 21.2% explained by the second component. Chroma was primarily influenced by TCC (0.52) and TOCC (0.52) followed by TYCC (0.47), all of which had positive loadings on the first component. On the other hand, TYCP (−0.33) indicated negative association with chroma.

The TA had a small positive effect on hue angle whilst it displayed a small negative influence on the chroma. TSS displayed the opposite effect, with a small negative impact on hue angle and a small positive influence on chroma. Both models indicated that TCC, TOCC, and TOCP played a significant role in hue and chroma. Higher TCC and TOCC were strongly associated with chroma, whilst TYCP resulted in contrasting influence on both colour measurements.

## 4. Discussion

The chroma and hue angle graph (Figure 1) illustrated a horn-like shape, with the variability of chroma increasing as the hue angle decreased. Other studies which report on the chroma and hue angle of fully ripened mangoes, also show that majority of their cultivars fell within the range of the samples in this study [5,6,26]. The few exceptions were still found to be confined within the flesh colour codes set by the Department of Agriculture and Fisheries (DAF). The colour rating used by DAF was also shown to be spread equally across the hue angle in ripened flesh in this study, but not the chroma range. It would appear there, that the subjective colour ratings that have previously been gathered by DAF (2015–2022) have been more related to hue angle than chroma.

In this study, TCC was one of the contributors to the difference in flesh colour in cultivars. A general trend of increasing orangeness was observed with an increase in TCC. Other studies also support this statement, with high carotenoid content within the mesocarp being reported to control both the hue angle and intensity of colour in multiple cultivars of mangoes [19,27]. However, this alone does not fully control the flesh colour as both the carotenoid proportions and concentrations have been shown in this study to influence the final flesh colour.

This was further supported by the PCA which showed that cultivars with higher TCC were more closely associated with increased chroma and lower hue angles. The clustering pattens observed in the PCA plot revealed that cultivars rich in orange carotenoids (e.g., all-*trans* β-carotene) aligned strongly with lower hue angles, while those with higher proportions of yellow carotenoids were positioned toward higher hue angles. The PCA also highlighted that while TCC is a key driver of chroma, the distribution of carotenoids also plays a major role in the final colouration. This was demonstrated in cv. Carrie, where despite its high TCC, it held high concentrations of orange and yellow carotenoids, resulting in a subjective colour rating of orange (4) indicating that carotenoid composition, rather than just TCC is vital for colour expression.

For example, whilst all-*trans* β-carotene is known for its orange pigmentation, α-carotene and lutein are known to be yellow and zeaxanthin as yellow-orange [9,13,28]. Furthermore, the λ_max_ of *cis*-isomers of β-carotene indicate subtle differences in colouration to the *trans*-isomer counterpart. The 9-*cis* and 13-*cis* isomer reflect more lighter and yellow colours respective to their difference in structure causing interruption of the conjugated double bonds [16].

In this study, cv. Carrie showed the highest TCC (14.55 mg/100 g DW) and exhibited the highest chroma and one of the lowest hue angles. On the other end of the spectrum, the lowest TCC was identified in cv. Ok Rong (0.196 mg/100 g DW). The proportion of carotenoids, specifically, all-*trans* β-carotene was an influencing factor in the final flesh colour. In cv. Carrie, despite having the highest TCC, the proportion of all-*trans* β-carotene was only 56%, compared to an average of 53.38%, with the highest proportion of all-*trans* β-carotene (68.88%) observed in cv. Creeper.

However, despite the difference in proportions, the concentration of all-*trans* β-carotene was still highest in cv. Carrie, which exhibited the highest TCC (8.1 mg/100 g DW), whilst cv. Creeper displayed 5.05 mg/100 g DW. In fact, cv. Creeper ranked sixth in the concentration ranking with cv. Carrie ranking first (Figure 2). The top four cultivars in terms of all-*trans* β-carotene concentration were the same as those ranked highest for TCC. This suggests that TCC plays a key role in determining the concentration of all-*trans* β-carotene, rather than just its proportion. This is consistent with the findings of Yungyuen et al. (2021), as well as observations in other β-carotene rich fruits and vegetables, including loquat and carrot [27,29,30].

In the cultivars with lower TCC, the proportions and concentrations of all-*trans* β-carotene displayed similar trends. Despite cv. Ok Rong exhibiting a higher β-carotene proportion of 38.2%, it only had a concentration of 0.196 mg/100 g DW, compared to cv. Xoai Than Ca, which exhibited a proportion of 27.3% and a concentration of 0.215 mg/100 g DW. This was interesting, as the pale-yellow pigmentation exhibited by cv. Xoai Than Ca was likely due to the combination of both the lower orange colouration influenced by the low all-*trans* β-carotene concentration, but also by the increased concentration of other carotenoids, particularly those with yellow colouration.

The importance of the proportions and concentrations, specifically of the principal carotenoid and orange-coloured carotenoids were strongly highlighted in the regression curve for the hue angle. The regression curve indicated an improvement in the R^2^ as the orange-coloured carotenoid concentration increased, and was not unexpected, as hue angle is a measurement of orangeness. TCC exhibited the lowest R^2^ value, as it includes carotenoids that are not strictly orange. Their inclusion led to an increase in hue angle, despite the substantial influence of all-*trans* β-carotene Overall, the importance of chroma is clearly associated with TCC, a relationship consistent with findings in watermelon, pumpkin, and squash [31,32]. However, hue angle appears to be more strongly influenced by the concentrations of specific carotenoids, particularly orange carotenoids, which play a key role in determining colour direction. This observation aligns with previous studies showing that hue angle is driven more by the accumulation of carotenoids such as β-carotene and β-cryptoxanthin, rather than by TCC alone [31,33]. For example, Itle and Kabelka (2009) reported a negative correlation between hue angle and β-carotene concentration in squash and pumpkin, where cultivars with lower hue angles exhibited higher levels of orange carotenoids [31].

This relationship was further validated using PLS regression, which quantified the influence of TCC and individual carotenoid concentrations on both hue angle and chroma. The first PLS component explained over 52.3% of the predictor variance for hue angle, confirming that TCC, TOCC and TOCP were the strongest contributors to colour differences. Similarly, the PLS model for chroma showed that TCC, TOCC and TYCC were positively correlated, reinforcing the role of total carotenoid concentration and increase in carotenoid concentration to enhance intensity of colour. Interestingly, TYCP was negatively associated with chroma, and its slight positive relationship with hue angle suggests that higher proportions of yellow carotenoids, without sufficient TCC, may contribute to more pale and yellow flesh colour in mangoes. This further supports the observation that the interaction between carotenoid profile and concentration is important in determining the final flesh colour.

**Figure 5:**
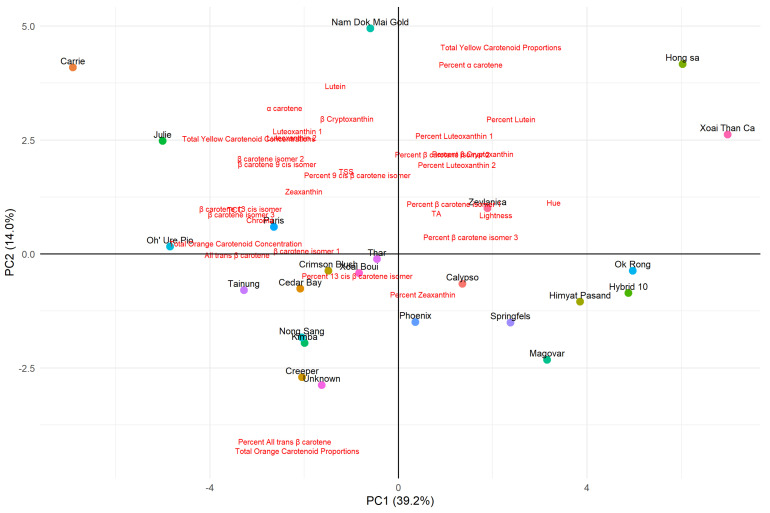
Principal component analysis (PCA) of 25 cultivars of mangoes showing a distribution of samples based on the first two principal components. The 25 cultivars are indicated by differently coloured circles with their respective cultivar names labelled above, whilst the measurement parameters measured in this study are indicated in red.

In this study, the 9-*cis* isomer and 13 *cis*-isomer of β-carotene were identified to have an almost identical maximum absorption as that of the yellow carotenoids, α-carotene and lutein. Consequently, it is likely these *cis*-isomers produce a similar colour of yellow. However, it was interesting to note that the more yellow-fleshed cultivars did not possess a higher proportion of these *cis*-isomers. Rather, it was the other yellow carotenoids (α-carotene, luteoxanthin isomers and lutein) which were observed to have a higher proportion. However, this may only be exclusive in cultivars with low TCC as cv. Carrie, despite being an orange cultivar, had high proportions of yellow carotenoids as seen in the PCA (Figure 5).

It is interesting to note that even with high proportions of yellow carotenoids, an increase in TCC can lead to darker and more orange colouration. This is further supported by cvs. Magovar and Springfels, which, despite having high proportions of orange carotenoids (67–70%), exhibit a yellow to pale-yellow coloration due to their low total carotenoid content. This highlights the critical role of total carotenoid concentration in determining fruit colour and intensity.

Another factor that may contribute to yellow colour development in certain cultivars is their apparent inability to fully ripen. Several green/pale-yellow cultivars had high TA and low TSS, which are common indicators of incomplete ripening in mango fruit [34]. The retardation of ripening may be linked to cultivar-specific differences in ethylene sensitivity. Ethylene is a key regulator of ripening, and variation in either ethylene production or responsiveness can influence post-harvest ripening speed [35,36,37,38]. However, variation in ethylene production is unlikely to be a factor in this study, as all cultivars harvested from the research station were treated with Ethrel^®^. Importantly, ethylene also regulates carotenoid biosynthesis by activating key genes in the pathway [39]. A lack of effective ethylene response in these cultivars potentially contributes to their low carotenoid concentrations, high TA and low TSS [27,40]. While this trend is not observed in all pale cultivars, a consistent pattern is apparent in several cases, suggesting that incomplete ripening may underlie some instances of poor colour development.

The mentioned inhibition of carotenoid biosynthesis is also reflected in the PLS and PCA models, where cultivars with low TCC clustered separately from those with deeper orange hues. The lower concentrations of all-*trans* β-carotene in these cultivars, coupled with high TA and low TSS, further supports the idea that incomplete ripening limits both the colour development and accumulation of carotenoids. These finding illustrate the importance of ripening physiology in determining flesh colour variation in mangoes.

## 5. Conclusions

This study demonstrates that both TCC and individual carotenoid levels influence mango flesh colour. Chroma was primarily driven by TCC, while hue angle was more closely linked to total orange carotenoid concentrations. While TCC played a significant role in influencing flesh colour, the proportions of orange and yellow carotenoids also contributed to the variation amongst cultivars. These findings offer insights for phytochemical breeding programmes aiming to enhance fruit quality by selecting for desirable colour traits. Future studies should also explore the role of ripening physiology, as several pale cultivars with low TCC showed high titratable acidity (TA) and low total soluble solids (TSS), suggesting that incomplete ripening may further limit colour development.

## Figures and Tables

**Table 1:** Proportions (%) of major carotenoids and minor carotenoids (grouped in ‘Other’, including β-carotene isomer 2, β-carotene isomer 3, zeaxanthin, lutein and β-cryptoxanthin) in the 25 mango cultivars. The subjective flesh colour codes are dark-orange (1), orange (2), orange-yellow (3), yellow (4) and green/pale-yellow (5).

Cultivar	All-*trans* β-Carotene (%)	13-*cis* β-Carotene (%)	9-*cis* β-Carotene (%)	α-Carotene (%)	Luteoxanthin Isomer (1) (%)	Luteoxanthin Isomer (2) (%)	Other (%)	Subjective Flesh Colour Code
Creeper	68.88	8.51	3.95	2.39	1.95	3.82	10.5	1
Unknown	67.81	8.47	4.57	1.40	1.59	2.84	13.32	1
Tainung	66.94	7.76	5.21	3.97	0.85	1.58	13.69	N/A
Nong Sang	66.66	6.98	5.57	2.44	2.68	4.15	11.52	1
Kimba	66.08	7.81	3.15	2.19	2.65	3.69	14.43	2
Phoenix	62.48	7.39	4.73	4.34	3.36	6.01	11.69	3
Cedar Bay	60.58	8.15	3.50	1.84	3.06	6.79	16.08	1
Xoai Boui	59.24	5.82	3.68	1.68	6.66	10.78	12.14	3
Oh’ Ure Pio	59.07	9.58	9.75	3.45	1.97	3.54	12.64	2
Paris	58.09	9.00	4.94	1.64	4.25	7.91	14.17	2
Springfels	55.71	6.87	5.85	2.18	3.17	6.98	19.24	4
Carrie	55.69	6.85	5.89	2.75	6.89	9.70	12.23	2
Crimson Blush	55.57	10.19	7.20	2.15	3.10	6.78	15.01	2
Magovar	55.47	8.25	10.21	1.92	3.63	7.17	13.35	5
Thar	54.28	10.66	7.15	4.55	4.07	6.54	12.75	3
Julie	54.08	10.56	16.11	4.47	1.30	2.31	11.17	1
Calypso^®^	53.14	9.37	4.30	2.07	6.52	11.83	12.77	2
Himayat Pasand	50.69	6.58	7.52	2.18	6.68	10.16	16.19	5
Hybrid 10	47.70	9.00	5.33	3.26	7.18	13.95	13.58	4
Zeylanica	46.85	7.93	5.05	2.70	6.91	12.52	18.04	5
Tung Chi	44.03	9.67	4.55	1.36	7.13	11.71	21.55	3
Ok Rong	38.43	11.51	4.76	3.82	2.84	4.92	33.72	4
Nam Dok Mai Gold	35.06	8.90	7.82	9.25	6.18	10.24	22.55	4
Xoai Than Ca	26.88	4.20	2.63	13.07	1.82	1.07	50.33	5
Hong Sa	25.01	6.74	6.81	9.39	8.14	14.24	29.67	4
LSD	3.61	1.18	1.09	0.96	1.31	2.27	2.85	

**Table 2:** Titratable Acidity (TA), Total Soluble Solids (TSS) and subjective flesh colour code for each of the 25 cultivars.

Cultivar	TA (g/100 g)	TSS (g/100 g)	TA: TSS Ratio	Subjective Flesh Colour Code
Oh’ Ure Pio	0.10	15.78	157.80	2
Xoai Boui	0.11	13.20	120.00	3
Hybrid 10	0.11	11.90	108.18	4
Hong Sa	0.12	17.72	147.67	4
Calypso^®^	0.13	13.00	100.00	2
Creeper	0.15	13.74	91.60	1
Nam Doc Mai Gold	0.16	18.30	114.38	4
Unknown	0.16	17.26	107.88	1
Himayat Pasand	0.17	16.82	98.94	5
Crimson Blush	0.18	19.95	110.83	2
Kimba	0.19	11.67	61.42	2
Tainung	0.19	16.45	86.58	NA
Springfels	0.19	11.67	61.42	4
Julie	0.21	18.03	85.86	1
Phoenix	0.21	12.42	59.14	3
Carrie	0.21	16.92	80.57	2
Cedar Bay	0.23	23.56	102.43	1
Thar	0.23	13.68	59.48	3
Ok Rong	0.28	18.68	66.71	4
Magovar	0.32	11.33	35.41	5
Zeylanica	0.32	18.35	57.34	5
Nong Sang	0.41	14.06	34.29	1
Paris	0.52	20.63	39.67	2
Xoai Than Ca	0.69	9.820	14.23	5
Tung Chi	NA	NA	NA	3
LSD	0.06	2.1		

**Table 3:** The partial least squares (PLS) regression loadings for hue angle and chroma.

Variables	Hue Angle (Loadings)	Chroma (Loadings)
Total Carotenoid Concentration (TCC)	−0.49	0.52
Total Orange Carotenoid Concentrations (TOCC)	−0.50	0.52
Total Orange Carotenoid Proportions (TOCP)	−0.39	0.33
Total Yellow Carotenoid Concentrations (TYCC)	−0.42	0.47
Total Yellow Carotenoid Proportions (TYCP)	0.39	−0.33
Titratable Acidity (TA)	0.11	−0.13
Total Soluble Solids (TSS)	−0.14	0.22

## Data Availability

Data from this manuscript is available upon request by contacting Tatsuyoshi Takagi.

## Abbreviations

The following abbreviations are used in this manuscript:

ANOVA	Analysis of Variance
BHT	Butylated Hydroxytoluene
CIE	Commission Internationale de l’Eclairage
DAF	Department of Agriculture and Fisheries
DCM	Dichloromethane
DW	Dry Weight
LSD	Least Significant Difference
MEP	Methylerythritol 4-Phosphate
MTBE	Methyl tert-Butyl Ether
PCA	Principal Component Analysis
PLS	Partial Least Squares
TA	Titratable Acidity
TCC	Total Carotenoid Concentration
TSS	Total Soluble Solids
TYCC	Total Yellow Carotenoid Concentrations
TYCP	Total Yellow Carotenoid Proportions
TOCC	Total Orange Carotenoid Concentrations
TOCP	Total Orange Carotenoid Proportions
UHPLC-DAD	Ultra-High Performance Liquid Chromatography with Diode-Array Detection

## References

[1] Lizada C. (1993). Mango. Biochemistry of Fruit Ripening.

[2] Singh Z., Singh R.K., Sane V.A., Nath P. (2013). Mango—Postharvest Biology and Biotechnology. Crit. Rev. Plant Sci..

[3] Baldevbhai P.J. (2012). Color Image Segmentation for Medical Images using L*a*b* Color Space. IOSR J. Electron. Commun. Eng..

[4] Manasa B., Jagadeesh S.L., Thammaiah N., Nethravathi (2019). Colour measurement of ripening mango fruits as influenced by pre-harvest treatments using L* a* b* coordinates. J. Pharmacogn. Phytochem..

[5] Gill P.P.S., Jawandha S.K., Kaur N. (2017). Transitions in mesocarp colour of mango fruits kept under variable temperatures. J. Food Sci. Technol..

[6] Vásquez-Caicedo A.L., Heller A., Neidhart S., Carle R. (2006). Chromoplast Morphology and β-Carotene Accumulation during Postharvest Ripening of Mango Cv. ‘Tommy Atkins’. J. Agric. Food Chem..

[7] Gomez-Lim M.A. (1997). Postharvest physiology. The mango: Botany, Production, and Uses.

[8] John J., Subbarayan C., Cama H.R. (1970). Carotenoids in 3 stages of ripening of mango. J. Food Sci..

[9] Natália M., Sandra R.S.F. (2016). Carotenoids Functionality, Sources, and Processing by Supercritical Technology: A Review. J. Chem..

[10] Clark S., Enna S.J., Bylund D.B. (2007). Beta Carotene. xPharm: The Comprehensive Pharmacology Reference.

[11] Mercadante A.Z., Rodriguez-Amaya D.B. (1998). Effects of Ripening, Cultivar Differences, and Processing on the Carotenoid Composition of Mango. J. Agric. Food Chem..

[12] Ben-Amotz A., Fishier R. (1998). Analysis of carotenoids with emphasis on 9-cis β-carotene in vegetables and fruits commonly consumed in Israel. Food Chem..

[13] Khoo H.-E., Prasad K.N., Kong K.-W., Jiang Y., Ismail A. (2011). Carotenoids and their isomers: Color pigments in fruits and vegetables. Molecules.

[14] Anusree M.K., Manasa Leela K., Sreehari M., Raj S., Sreenikethanam A., Bajhaiya A.K., Meena S.N., Nandre V., Kodam K., Meena R.S. (2023). Chapter 14—Marine microalgae: An emerging source of pharmaceuticals and bioactive compounds. New Horizons in Natural Compound Research.

[15] Meléndez-Martínez A.J., Britton G., Vicario I.M., Heredia F.J. (2007). Relationship between the colour and the chemical structure of carotenoid pigments. Food Chem..

[16] Rodriguez-Amaya D.B., Kimura M. (2004). HarvestPlus Handbook for Carotenoid Analysis.

[17] Rodriguez-Amaya D.B., Esquivel P., Meléndez-Martínez A.J. (2023). Comprehensive Update on Carotenoid Colorants from Plants and Microalgae: Challenges and Advances from Research Laboratories to Industry. Foods.

[18] Jing C., Qun X., Rohrer J. (2012). HPLC Separation of All-Trans-β-Carotene and Its Iodine-Induced Isomers Using a C30 Column. Thermo. Sci..

[19] Vásquez-Caicedo A.L., Sruamsiri P., Carle R., Neidhart S. (2005). Accumulation of All-trans-β-carotene and Its 9-cis and 13-cis Stereoisomers during Postharvest Ripening of Nine Thai Mango Cultivars. J. Agric. Food Chem..

[20] Britton G., Liaaen-Jensen S., Pfander H. (2004). Carotenoids Handbook.

[21] Simpson K., Quiroz L.F., Rodriguez-Concepción M., Stange C.R. (2016). Differential Contribution of the First Two Enzymes of the MEP Pathway to the Supply of Metabolic Precursors for Carotenoid and Chlorophyll Biosynthesis in Carrot (Daucus carota). Front. Plant Sci..

[22] Galpaz N., Wang Q., Menda N., Zamir D., Hirschberg J. (2008). Abscisic acid deficiency in the tomato mutant high-pigment 3 leading to increased plastid number and higher fruit lycopene content. Plant J..

[23] Lu S., Van Eck J., Zhou X., Lopez A.B., O’Halloran D.M., Cosman K.M., Conlin B.J., Paolillo D.J., Garvin D.F., Vrebalov J. (2006). The Cauliflower Or Gene Encodes a DnaJ Cysteine-Rich Domain-Containing Protein That Mediates High Levels of β-Carotene Accumulation. Plant Cell.

[24] British Colour Council (1942). Horticultural Colour Chart. Issued by the British Colour Council in Collaboration with the Royal Horticultural Society.

[25] Hong H.T., Takagi T., O’Hare T.J. (2022). An optimal saponification and extraction method to determine carotenoids in avocado. Food Chem..

[26] Thinh D.C., Uthaibutra J., Joomwong A. (2013). Effect of storage temperatures on ripening behavior and quality change of vietnamese mango cv. Cat Hoa Loc. Int. J. Biotechnol. Res..

[27] Yungyuen W., Thi Thuong V., Apiradee U., Gang M., Lancui Z., Nopparat T., Samak K., Pongphen J., Masaya K. (2021). Carotenoid Accumulation and the Expression of Carotenoid Metabolic Genes in Mango during Fruit Development and Ripening. Appl. Sci..

[28] Minguez-Mosquera M.I., Grandul-Rojas B., Garrido-Fernandez J., Gallardo-Guerrero L. (1990). Pigments present in virgin olive oil. J. Am. Oil Chem. Soc..

[29] Rodriguez-Concepcion M., Stange C. (2013). Biosynthesis of carotenoids in carrot: An underground story comes to light. Arch. Biochem. Biophys..

[30] Zhou C.-H., Xu C.-J., Sun C.-D., Li X., Chen K.-S. (2007). Carotenoids in White- and Red-Fleshed Loquat Fruits. J. Agric. Food Chem..

[31] Itle R.A., Kabelka E.A. (2009). Correlation Between Lab Color Space Values and Carotenoid Content in Pumpkins and Squash (*Cucurbita* spp.). HortScience.

[32] Yuan P., Umer M.J., He N., Zhao S., Lu X., Zhu H., Gong C., Diao W., Gebremeskel H., Kuang H. (2021). Transcriptome regulation of carotenoids in five flesh-colored watermelons (*Citrullus lanatus*). BMC Plant Biol..

[33] Zhou J.-Y., Sun C.-D., Zhang L.-L., Dai X., Xu C.-J., Chen K.-S. (2010). Preferential accumulation of orange-colored carotenoids in Ponkan (*Citrus reticulata*) fruit peel following postharvest application of ethylene or ethephon. Sci. Hortic..

[34] Padda M.S., do Amarante C.V.T., Garcia R.M., Slaughter D.C., Mitcham E.J. (2011). Methods to analyze physico-chemical changes during mango ripening: A multivariate approach. Postharvest Biol. Technol..

[35] Ma J.-H., Li W., Chen M.-M., Zeng J.-K. (2021). Effects of different treatments on texture of postharvest mango fruits. Storage Process.

[36] Montalvo E., García H.S., Tovar B., Mata M. (2007). Application of exogenous ethylene on postharvest ripening of refrigerated ‘Ataulfo’ mangoes. Food Sci. Technol..

[37] Alos E., Martinez-Fuentes A., Reig C., Mesejo C., Zacarías L., Agustí M., Rodrigo M.J. (2019). Involvement of ethylene in color changes and carotenoid biosynthesis in loquat fruit (*Eriobotrya japonica* Lindl. cv. Algerie). Postharvest Biol. Technol..

[38] Abbas M.e., Fandi B.S. (2002). Respiration rate, ethylene production and biochemical changes during fruit development and maturation of jujube (*Ziziphus mauritiana* Lamk). J. Sci. Food Agric..

[39] Cruz A.B., Bianchetti R.E., Rodrigues Alves F.R., Purgatto E., Pereira Peres L.E., Rossi M., Freschi L. (2018). Light, ethylene and auxin signaling interaction regulates carotenoid biosynthesis during tomato fruit ripening. Front. Plant Sci..

[40] Saeed M., Zhao L., Rashwan A.K., Osman A.I., Chen Z., Wang G., Zhou C., Tu T., Alabd A., Jiao Y. (2024). Ethylene-Induced Postharvest Changes in Five Chinese Bayberry Cultivars Affecting the Fruit Ripening and Shelf Life. Horticulturae.

